# Supplementary figures and images for: Aquatic insects dealing with dehydration: do desiccation resistance traits differ in species with contrasting habitat preferences?
Source: PeerJ. 2016 Aug 31;4:e2382. doi: 10.7717/peerj.2382 (PMC5012287; doi:10.7717/peerj.2382)

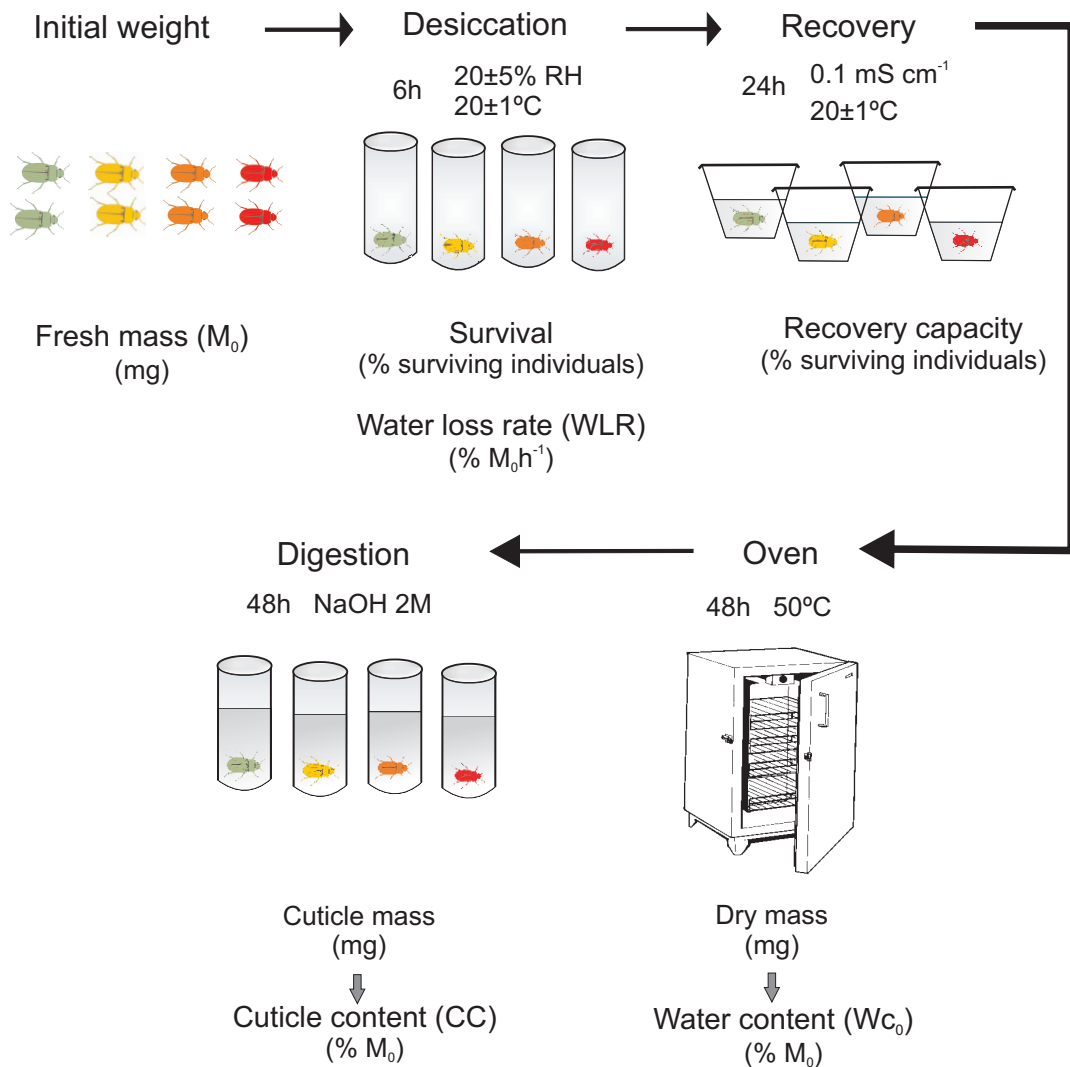

Supplement: Figure S1 [file peerj-04-2382-s001.pdf]
