# Supplementary material for: Aquatic insects dealing with dehydration: do desiccation resistance traits differ in species with contrasting habitat preferences?
Source: PeerJ. 2016 Aug 31;4:e2382. doi: 10.7717/peerj.2382 (PMC5012287; doi:10.7717/peerj.2382)
Supplement: Table S1 [file peerj-04-2382-s002.docx]

**Table S.2** Summary of variation in desiccation resistance traits in control and treatment groups of *Enochrus* species.

|  |  | N | Initial fresh mass (mg) | | | Initial water content  (% of fresh mass) | | | Cuticle content  (% of fresh mass) | | | Total water loss  (% of water content) | | | Water loss rate  (% of fresh mass per hour) | | |
| --- | --- | --- | --- | --- | --- | --- | --- | --- | --- | --- | --- | --- | --- | --- | --- | --- | --- |
| Control group | |  |  |  |  |  |  |  |  |  |  |  |  |  |  |  |  |
|  | *E. halophilus* | 10 | 8.703 | ± | 1.528 | 60.6 | ± | 2.7 |  |  |  | 5.9 | ± | 1.1 | 0.51 | ± | 0.32 |
|  | *E. politus* | 20 | 10.239 | ± | 1.258 | 62.5 | ± | 3.0 |  |  |  | 6.1 | ± | 0.5 | 0.67 | ± | 0.25 |
|  | *E. bicolor* | 15 | 10.175 | ± | 1.274 | 66.4 | ± | 3. 3 |  |  |  | 4.3 | ± | 0.5 | 0.48 | ± | 0.25 |
|  | *E. jesusarribasi* | 20 | 7.211 | ± | 1.174 | 65.3 | ± | 2.4 |  |  |  | 4.3 | ± | 0.3 | 0.48 | ± | 0.15 |
| Treatment group | |  |  |  |  |  |  |  |  |  |  |  |  |  |  |  |  |
|  | *E. halophilus* | 17 | 8.035 | ± | 1.288 | 62. 3 | ± | 3.5 | 22.3 | ± | 3.5 | 39.1 | ± | 2.9 | 3.57 | ± | 1.12 |
|  | *E. politus* | 20 | 10.439 | ± | 1.252 | 62.4 | ± | 2.3 | 15.9 | ± | 2.3 | 24.8 | ± | 1.1 | 2.59 | ± | 0.59 |
|  | *E. bicolor* | 20 | 9.784 | ± | 1.024 | 68.3 | ± | 2.2 | 14.0 | ± | 2.6 | 19.3 | ± | 1.1 | 2.22 | ± | 0.60 |
|  | *E. jesusarribasi* | 20 | 7.048 | ± | 1.304 | 66.3 | ± | 3.2 | 12.6 | ± | 2.3 | 20.0 | ± | 0.6 | 2.28 | ± | 0.40 |

Data are reported as mean ± SD. N, sample size.
